# Oral manifestations of patients with systemic sclerosis: a meta-analysis for case-controlled studies

**DOI:** 10.1186/s12903-021-01603-2

**Published:** 2021-05-10

**Authors:** Suhan Zhang, Junfei Zhu, Yanshan Zhu, Xiaochao Zhang, Ruifang Wu, Siying Li, Yuwen Su

**Affiliations:** 1 Department of Dermatology, Hunan Key Laboratory of Medical Epigenomics, The Second Xiangya Hospital of Central South University, No.139 Middle Renmin road, Changsha, China; 2 Stomatology Center of China Japan Friendship Hospital, Beijing, China

**Keywords:** Oral hygiene, Periodontitis, Periodontal-systemic disease interactions

## Abstract

**Background:**

Systemic sclerosis (SSc) is a multisystem rheumatic disease. Orofacial manifestations are commonly in SSc but maybe usually ignored and overshadowed by other systemic complications. Multiple comparative studies have been conducted to investigate the possible links between SSc and oral manifestations. The present study aimed to investigate the oral health status in patients with SSc.

**Methods:**

Pubmed, Embase, Web of Science, and Scopus were searched up to July 2020. Following outcomes were evaluated: Probing depth (PD), Attachment loss (AL), Bleeding on probing (BOP), Number or percentage of Sites with PD ≥ 4 mm, Prevalence of periodontitis, Number of teeth, Decayed Teeth, Missing teeth, Filled teeth, DMFT index, and the interincisal distance. Newcastle-Ottawa Scale (NOS) were applied for quality assessment. The statistical analysis was processed using the software STATA.

**Results:**

11 eligible studies were included. The maximum interincisor distance was significantly restricted in SSc patients (SMD − 1.061; 95 %CI [− 1.546, − 0.576]; Z = 4.29, *P* = 0.000).The prevalence of Periodontitis (OR 7.007; 95 %CI [3.529, 13.915]; Z = 5.56, *P* = 0.000), PD (SMD 3.101; 95 %CI [1.374, 4.829]; Z = 3.52, *P* = 0.000), AL(SMD 2.584; 95 %CI [0.321, 4.846]; Z = 2.24, *P* = 0.025), sites with PD ≥ 4mm (SMD 2.071 ; 95 %CI [0.267, 3.875]; Z = 2.25, *P* = 0.024) and the number of decayed teeth (SMD, 0.186; 95 %CI [0.007, 0.365]; Z = 2.04, *P* = 0.041) were increased significantly in SSc population in comparison with the controls.

**Conclusions:**

SSc patients have limited mouth opening, higher periodontitis prevalence, and worse periodontal status, as well as an increased number of decayed teeth. Routinely oral hygiene instruction and initial periodontal treatment is recommended for SSc patients.

**Supplementary Information:**

The online version contains supplementary material available at 10.1186/s12903-021-01603-2.

## Introduction

Systemic sclerosis (SSc) is a multisystem rheumatic disorder involving the skin, connective tissue, and internal organs. SSc is characterized by autoimmunity activation, small arteries vasculopathy, and multi-organ fibrosis. Due to complex complication, SSc entails high mortality, with an overall SMR of 2.72 [1]. Additionally, there is also a mass of non-lethal burden that leads to a range of severe and disabling symptoms that impacts the quality of life [2].

Oral diseases, especially periodontitis, have been reported to be connected with multiple systemic pathological changes such as diabetes, coronary heart diseases, as well as autoimmune rheumatic diseases [3–6]. Likewise, Orofacial manifestations are commonly in SSc (up to 80 %) but may be usually ignored and overshadowed by other systemic complications [7]. The onset of oral disorders in SSc is associated with the reduced mouth opening (microstomia) and decreased salivary flow (xerostomia), which could lead to several dental and periodontal disorders such as dental caries, gingivitis as well as periodontitis [8]. In 2018, Rawen et al. firstly published a systematic review aiming to study the influence of SSc oral manifestations on patients’ health-related quality of life, and the conclusion indicated an impaired quality of life in the SSc patients caused by the oropharyngeal dysfunctions. However, because of the rarity of SSc, large-scale studies and quantitative analysis were still needed [9].

From the existed literature, it was deduced that the chronic inflammation secondary to autoimmune damage was related to the etiology of SSc [10]. Identically, the onset of oral disorders, such as dental caries and periodontitis, were also considered to be involved with inflammatory factors [11]. The biological plausibility for the association between SSc and oral disorders has been revealed by studies [12]. In recent years, comparative studies have been conducted to investigate the possible links between SSc and oral manifestations, however, the findings were inconsistent [7, 13–16]. Thereby, the present meta-analysis inclined to quantitatively investigate an SSc-Oral association through evidence-based methods. The aim of present study was to evaluate the association between oral manifestations and the presence of SSc, compared to the SSc-free populations.

## Methods

The study was designed under the following PECO framework: Population: Any population; Exposure: Exposed to SSc; Comparator: Not exposed to SSc; Outcomes: Oral manifestations. A search of the literature was conducted through Pubmed, Embase, Web of Science, and Scopus to July 2020. The search strategies were listed in the Additional file 1: Table S1. The Preferred Reporting Items for Systematic Review and Meta-analyses (PRISMA) statements were followed [17].

There is no clinical intervention, body samples collection, and privacy disclosure of patients in the current study. Therefore, approval from ethical boards and informed consent are not required.

### Eligibility criteria

The inclusion criteria were: (1) Case-control studies; (2) The patients in the case groups should be diagnosed with SSc and the controls were SSc-free individuals; (3) The result of the study should include one of the following outcomes: Probing depth (PD), Attachment loss (AL), Bleeding on probing (BOP), Number or percentage of Sites with PD ≥ 4 mm, Prevalence of periodontitis, Number of teeth, Decayed Teeth, Missing teeth, Filled teeth, DMFT index, and the interincisal distance.

The exclusion criteria included: (1) Review, retrospective studies, case reports, comments or conference abstracts; (2) Studies with insufficient data for the statistical analysis; (3) Studies with repeated data.

### Record screen

The authors screened the title and abstract of the initially obtained literatures according to the eligibility criteria, and the full-text paper was screened for a further selection. Three authors were involved: the screen of searching records was conducted by two independent authors (Suhan Zhang and Junfei Zhu), and another author (Yuwen Su) was consulted to reach a consensus.

### Data extraction

The data extraction process was conducted by two authors (Suhan Zhang and Junfei Zhu). The following demographic data and information were extracted: (1) First author; (2) Publication Year; (3) Country; (4) Group size; (5) Gender (6) Age, and (7) Outcomes;

### Risk of bias assessment

The methodological quality was assessed by two authors (Suhan Zhang and Junfei Zhu), the discrepancies were resolved through discussion. The authors applied the Newcastle-Ottawa Scale (NOS) to evaluate the quality of the included case-control studies. NOS was composited by 3 chapters, which are “Selection” (0–4 points), “Comparability” (0–2 points), and “Exposure” (0–3 points). The total scores were summed up. Studies with scores of 0–3, 4–6, and 7–9 points were considered low, moderate, and high quality, respectively[18].

### Meta-analysis

The data of the continuous variable was presented in mean (M) ± standard deviation (SD), and the standard mean difference (SMD) and corresponding 95 % confidence interval (CI) were used. If the data was given by the median (minimum-maximum), the estimation method recommended by Hozo et al. [19] was used for transformation. For dichotomous variables, odds ratio (OR) and CIs were calculated to evaluate the prevalence of periodontitis. Heterogeneity was evaluated by chi-square and I^2^; a *P* value < 0.05 and I^2^ > 50 % were considered as a significant heterogeneity then the random-effect model was used; otherwise, the fixed-effect model was employed to assure the statistical efficiency. The statistical analysis was processed using the software STATA 12.0.

## Results

### Characteristics and quality assessment

**Fig. 1:**
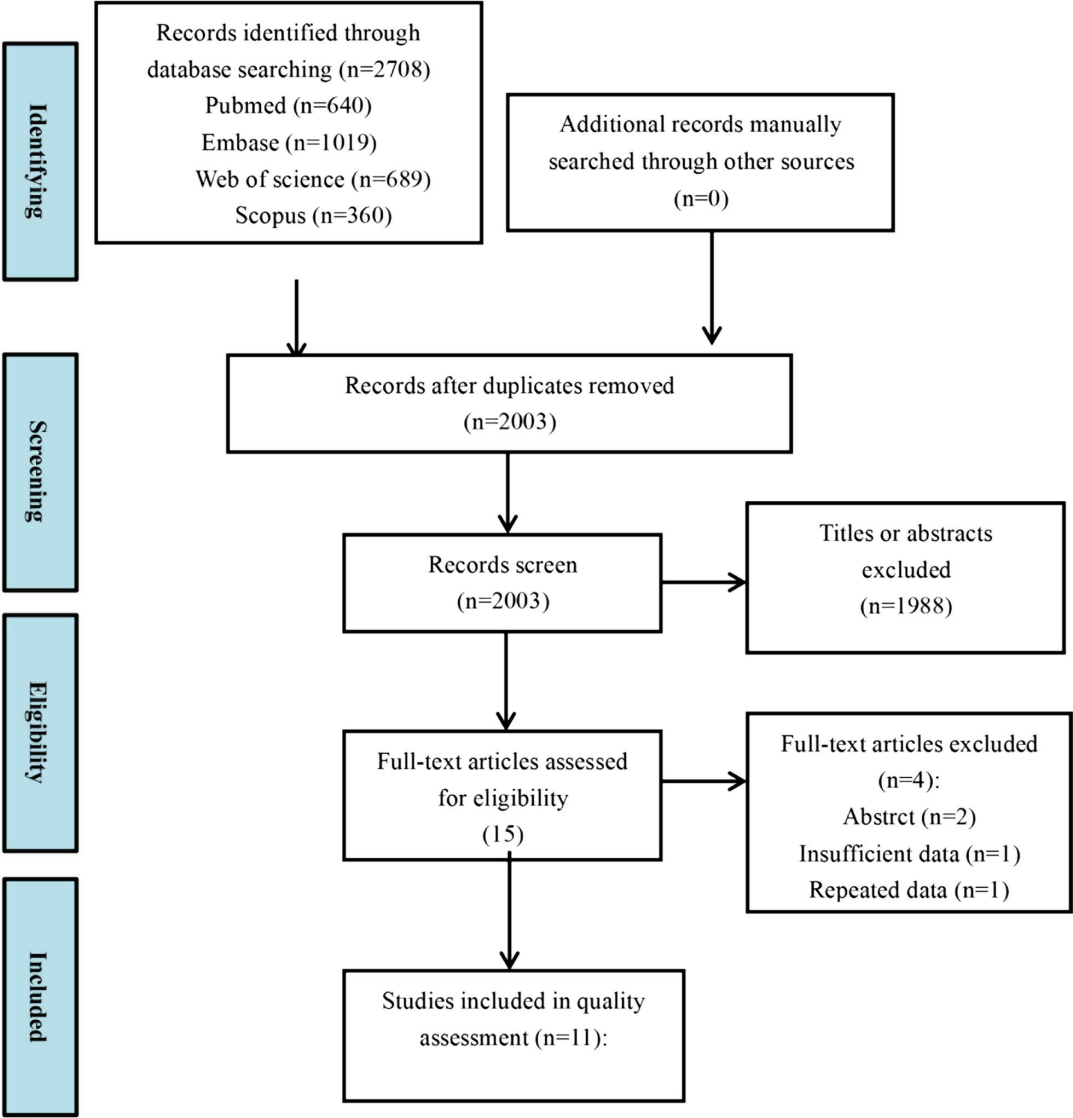
Flow chart of study selection

2708 potentially relevant records were identified through the search. After the title and abstract screen, 15 studies were eligible for full-text review. Finally, a total of 11 eligible studies were included [7, 13–16, 20–25] (Fig. 1). All the included studies employed case-control designs. 4 of the studies were from Asia [14, 16, 21, 23], 4 were from America [13, 15, 22, 25]. and 3 were from Europe [7, 20, 24]. The NOS score of the studies ranged from 4 to 8. 3 studies were considered of moderate quality [14, 15, 22], and 8 studies were high-qualitied [7, 13, 16, 20, 21, 23–25]. The characteristics and quality assessment results were presented in Tables 1 and 2.

**Table 1:** Charicteristics of the included studies

Author and year	Country	Design	Sample size (SS/CTR)	Age (SS/CTR)	Female (SS/CTR)	Outcomes for meta-analysis
Baron 2014	Canada	Case-controlled	163/231	56.2/58.1 (*P* > 0.05)	146/209 (*P* > 0.05)	Decayed teeth/ Missing teeth/ Filled teeth/Interincisal distance
Chu 2011	China	Case-controlled	40/40	(*P* > 0.05)	(*P* > 0.05)	Decayed teeth/ Missing teeth/ Filled teeth/DMFT/Interincisal distance/Prevalence of periodontitis/Number of teeth
da Silva 2019	Brazil	Case-controlled	50/43	46/44	43/36	PD/AL/Prevalence of periodontitis
Elimelech 2015	Israel	Case-controlled	20/20	45.36/48.5 (P > 0.05)	18/15	PD/BOP/Sites with PD > 4mm
Iordache 2019	Romania	Case-controlled	43/43	43.95/NA (*P* > 0.05)	31/NA (*P* > 0.05)	PD/Number of teeth
Isola 2017	Italy	Case-controlled	54/55	48.7/47.3 (*P* > 0.05)	36/30 (*P* > 0.05)	PD/AL/BOP/Sites with PD > 4mm/Missing teeth
Leung 2011	China	Case-controlled	36/36	35/NA (*P* > 0.05)	50.6/NA (*P* > 0.05)	PD/AL/BOP/Number of teeth
Marcucci 2009	Brazil	Case-controlled	15/10	43.72/31.82	13/NA	Interincisal distance
Mayer 2013	Israel	Case-controlled	12/12	48.4 /51.5 (*P* > 0.05)	9/7 (*P* > 0.05)	PD/BOP/Sites with PD > 4mm
Pischon 2016	Germany	Case-controlled	58/52	55.1/52.1(*P* > 0.05)	44/43 (*P* > 0.05)	PD/AL/BOP/DMFT/Missing teeth/Number of teeth
Wood 1988	Canada	Case-controlled	31/30	51.9/50.3 (*P* > 0.05)	(*P* > 0.05)	Interincisal distance/DMFT

*SS*, Systemic sclerosis, *CTR* control

**Table 2:** Quality assessment

Author and year	Selection				Comparability		Exposure			Total
Baron 2014	●	●	○	●	●	●	○	●	●	7
Chu 2011	○	○	●	●	●	●	○	●	●	6
da Silva 2019	●	○	●	●	●	●	○	○	●	6
Elimelech 2015	●	●	●	●	●	●	○	●	●	8
Iordache 2019	●	●	○	●	●	●	○	●	●	7
Isola 2017	●	○	●	●	●	●	○	●	●	7
Leung 2011	●	○	●	●	●	●	●	●	●	8
Marcucci 2009	●	○	○	●	○	○	○	●	●	4
Mayer 2013	●	●	●	●	●	●	○	●	●	8
Pischon 2016	●	○	●	●	●	●	○	●	●	7
Wood 1988	●	○	●	●	●	●	○	●	●	7

## Oral manifestations

**Fig. 2:**
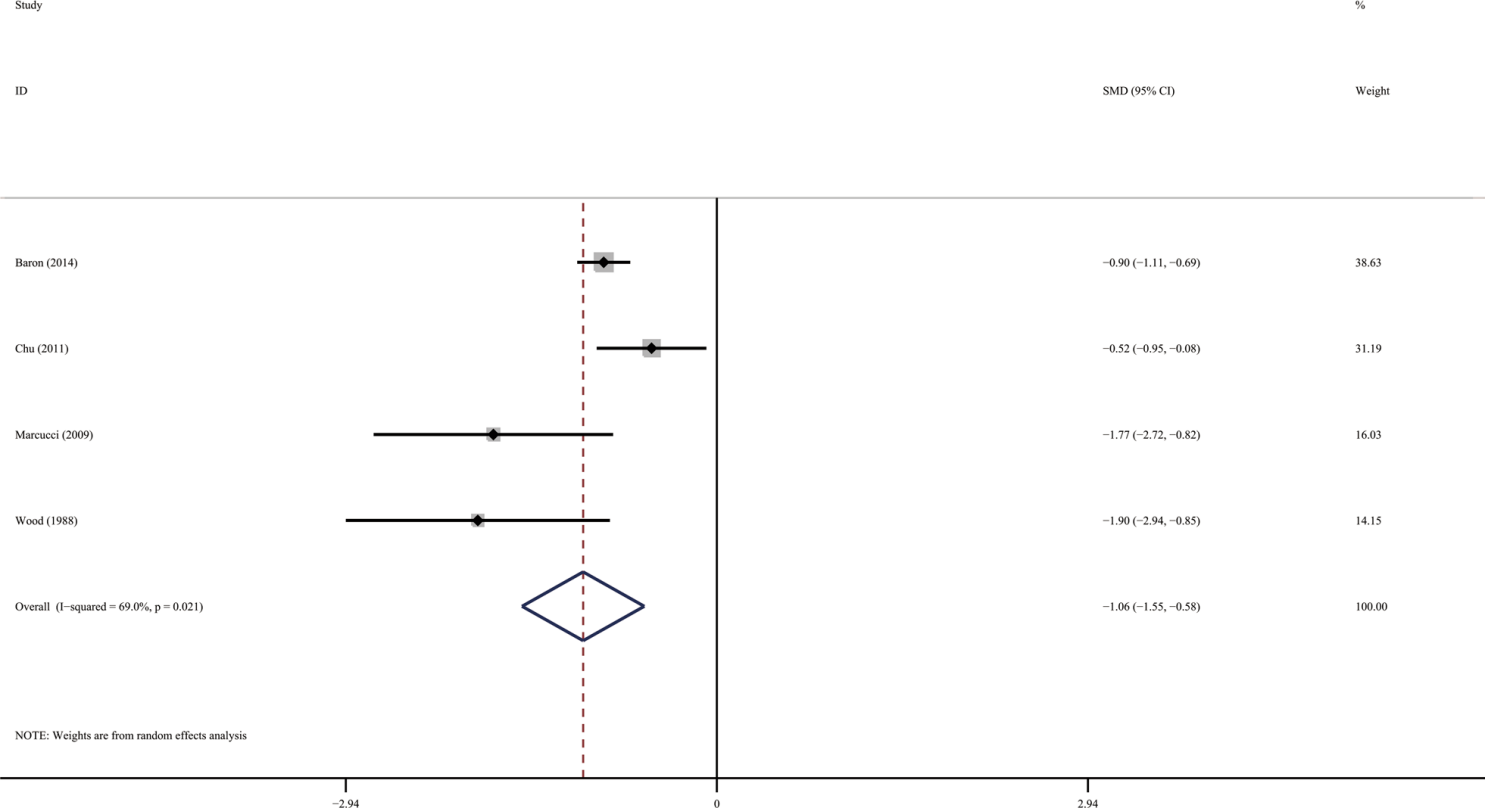
Forest plots of the maximum interincisor distance (Mouth opening)

The maximum interincisor distance (Mouth opening) was significantly restricted in SSc patients compared with the controls (Fig. 2; SMD − 1.061; 95 %CI [− 1.546, − 0.576]; Z = 4.29, *P* = 0.000; Heterogeneity: I^2^ = 69.0 %, *P* = 0.021).

**Fig. 3:**
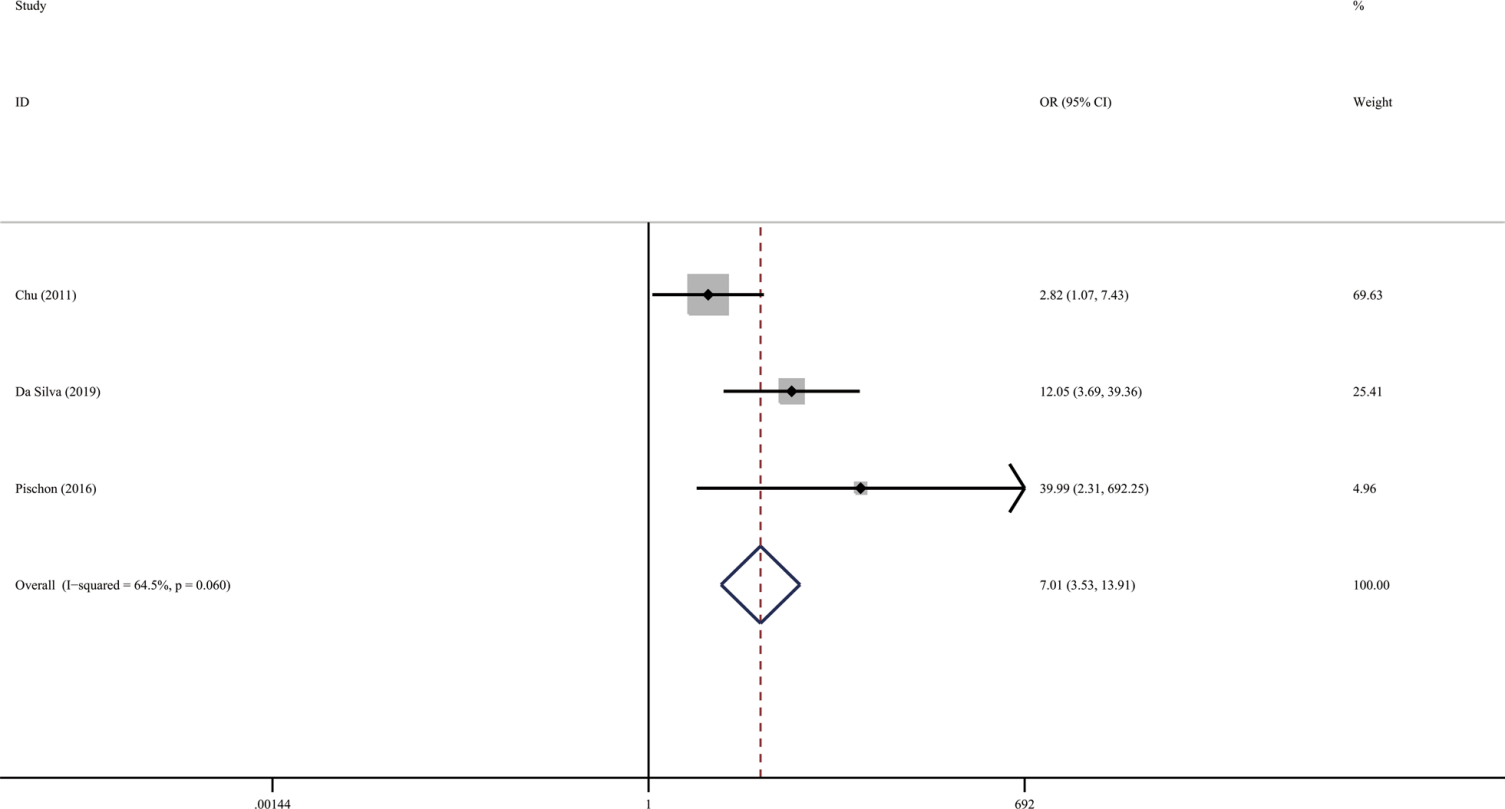
Forest plots of the prevalence of periodontitis

**Fig. 4:**
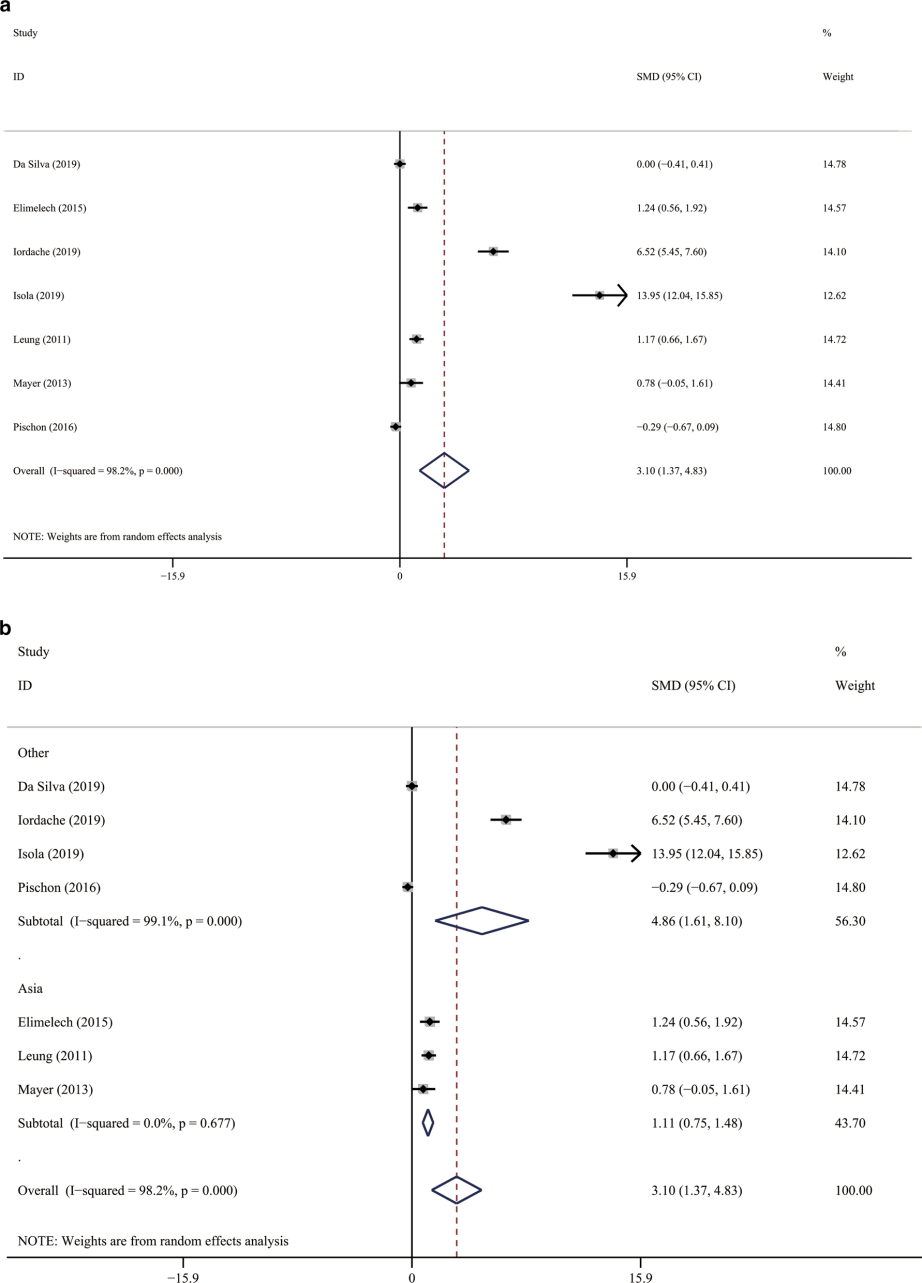
Forest plots of the PD. **a** General analysis; **b** subgroup analysis

**Fig. 5:**
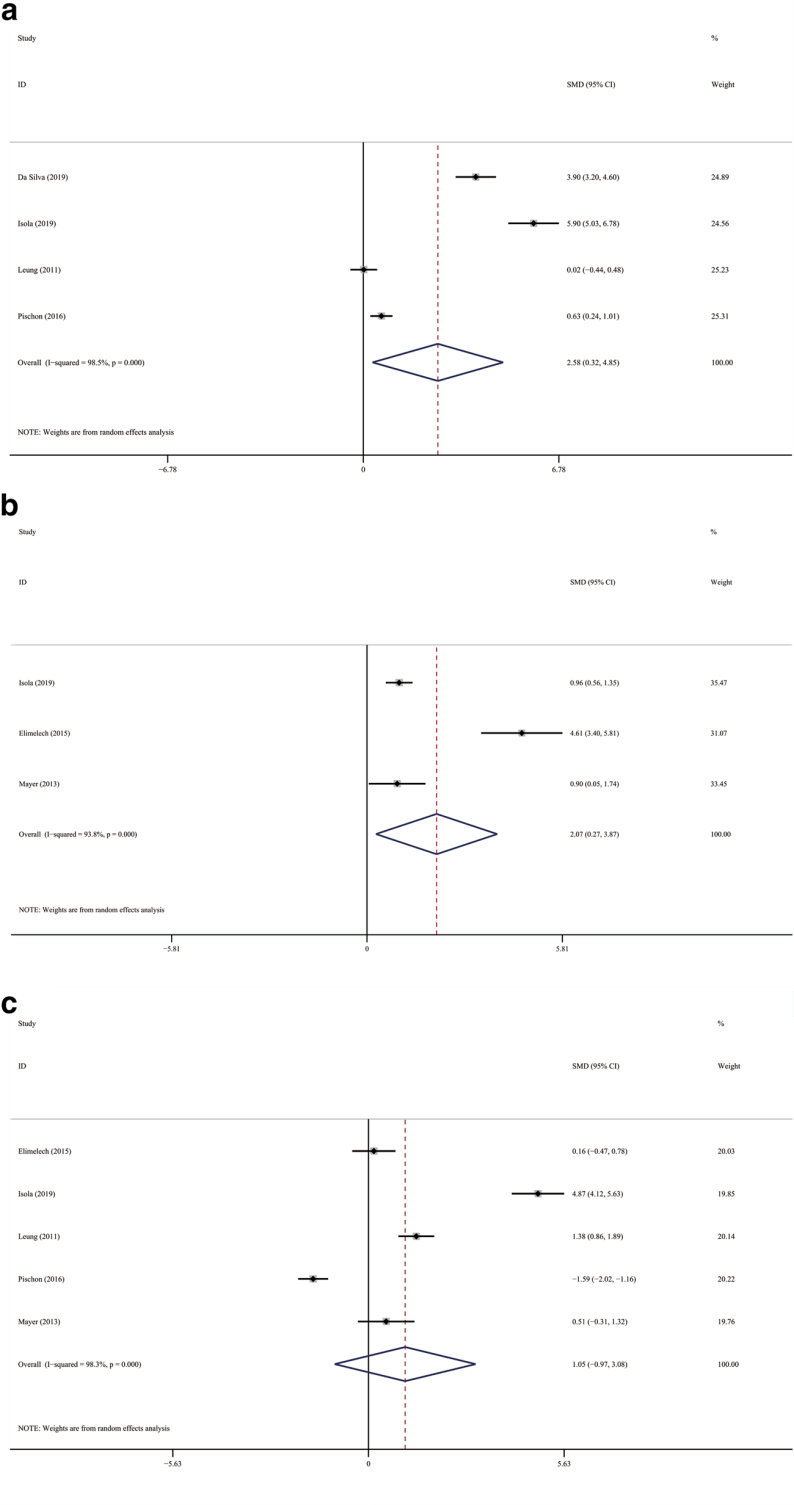
Forest plots of other periodontal parameters. **a** AL; **b** sites with PD ≥ 4mm; **c** BOP

The result of meta-analysis found a significantly increased periodontitis prevalence in the SSc population. (Fig. 3; OR 7.007; 95 %CI [3.529,13.915]; Z = 5.56, *P* = 0.000; Heterogeneity: I^2^ = 64.5 %, *P* = 0.060). PD was significantly elevated in SSc patients in comparison with the controls (Fig. 4a; SMD 3.101; 95 %CI [1.374, 4.829]; Z = 3.52, *P* = 0.000; Heterogeneity: I^2^ = 98.2 %, *P* = 0.000.). We used subgroup analysis to elucidate the heterogeneity. When the analysis was stratified by nation, the Asia subgroup demonstrated an insignificant heterogeneity (Fig. 4b; Asia: SMD1.113; 95 %CI [0.751,1.476]; Z = 6.01, *P* = 0.000; Heterogeneity: I^2^ = 0, *P* = 0.677). In accordance with PD, SSc patients have significantly increased Al and more sites with PD ≥ 4mm compared with the controls. (Fig. 5a; AL: SMD 2.584; 95 %CI [0.321, 4.846]; Z = 2.24, *P* = 0.025; Heterogeneity: I^2^ = 98.5 %, *P* = 0.000; Fig. 5b sites with PD ≥ 4mm: SMD 2.071 ; 95 %CI [0.267, 3.875]; Z = 2.25, *P* = 0.024; Heterogeneity: I^2^ = 93.8 %, *P* = 0.000). Additionally, statistical significance was not found in the meta-analysis regarding to the BOP (Fig. 5c; SMD, 1.054; 95 %CI [− 0.973, 3.081]; Z = 2.24, *P* = 0.025; Heterogeneity: I^2^ = 98.5 %, *P* = 0.000).

**Fig. 6:**
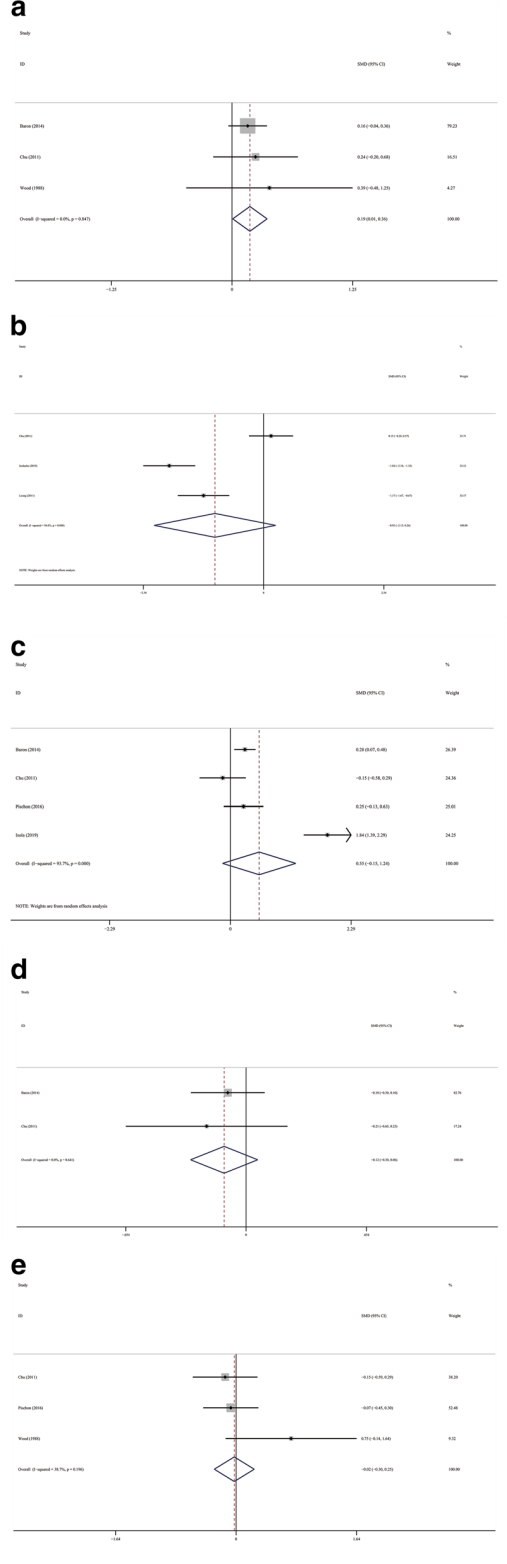
Forest plots of other parameters. **a** Decayed teeth; **b** Number of teeth; **c** Missing teeth; **d** Filled teeth; **e** DMFT

SSc patients have significantly increased number of decayed teeth than the controls (Fig. 6a; SMD, 0.186; 95 %CI [0.007, 0.365]; Z = 2.04, *P* = 0.041; Heterogeneity: I^2^ = 0 %, *P* = 0.847). The significant results of the number of teeth, missing teeth, filled teeth and DMFT index were not observed between the groups. (Fig. 6b; Number of teeth: SMD − 0.947; 95 %CI [− 2.131, 0.237]; Z = 1.57, *P* = 0.117; Heterogeneity: I^2^ = 94.6 %, *P* = 0.000; Fig. 6c missing teeth: SMD 0.546; 95 %CI [− 0.148, 1.241]; Z = 1.54, *P* = 0.123; Heterogeneity: I^2^ = 93.7 %, *P* = 0.000; (Fig. 6d filled teeth: SMD − 0.119; 95 %CI [− 0.302, 0.063]; Z = 1.28, *P* = 0.200; Heterogeneity: I^2^ = 0 %, *P* = 0.641; Fig. 6e DMFT: SMD − 0.025; 95 %CI [− 0.296, 0.247]; Z = 0.18, *P* = 0.858; Heterogeneity: I^2^ = 38.7 %, *P* = 0.169)

## Discussion

The present study aimed to assess the oral health status in SSc patients compared with healthy individuals. The results of the meta-analysis indicated that SSc patients have limited mouth opening and worse periodontal status compared with the healthy populations.

The most factor associated SSc and oral manifestations together were the reduced mouth opening, which was caused by the sclerosis of skin and masticatory muscles. The extent of the mouth opening restriction was correlated with various variables, such as gender and disease severity[26]. It was certain that the restriction of mouth opening will affect the quality of lives of SSc patients mostly because of weakened eating abilities and the difficulties in oral hygiene maintenance[27]. According to our findings, SSc patients tend to have higher periodontitis prevalence, and the elevation of periodontal parameters, such as PD and AL were detected in SSc populations. One of the reasons for the declined periodontal status in SSc patients is the restricted mouth opening, which makes it difficult for the patients to maintain daily oral hygiene and for the dentists to conduct the oral exam and dental treatment. Besides, SSc always developed accompanied by the Xerostomia. It was reported that 14 % of the patients might have secondary Sjögren’s syndrome [28]. The insufficient salivary flow decreased the self-clean ability of the oral cavity and impaired the buffer balance, which makes a beneficial situation for bacterial colonization, and contributes to periodontal and dental pathology [29]. Additionally, Esophageal reflux, another concomitant symptom of SSc patients, raised oral cavity acidity and also contributes to the growth of periodontal pathogens [30].

Periodontitis is an inflammatory disorder raised by the accumulation of specific bacterial pathogens and the destruction of host immune responses [31]. The clinical findings of periodontitis included gingival bleeding, tooth mobility, and finally the loss of the tooth, which are associated with a negative impact on Oral Health Related Quality of Life (OHRQoL) [32]. Similarities could be discovered between periodontitis and SSc. Studies suggested that several pathomechanisms might be shared by these two chronic diseases, one of which is the elevated circulating inflammatory biomarkers, such as C-reactive protein (CRP), Tumor Necrosis Factor (TNF)-α, IL(Interleukin)-6, 1, and 17 [16]. The study conducted by Ozcelik et al. showed that the gingival infiltration and microvessel density were elevated in the gingiva samples of SSc patients, however, the vascular endothelial growth factor (VEGF) expressions were decreased, suggesting vessel degeneration [1]. The vascular alterations might inhibit the angiogenic responsiveness to bacteria plaque in periodontal tissues [33], which accelerated the destruction of periodontal ligaments and the surrounding alveolar bones, presenting with increased PD and AL. Moreover, in the researches regarding bone metabolism, the level of soluble receptor activator for nuclear factor kappa beta ligand (RANKL) was reported to be elevated in SSc patients, which was related to osteoporosis in SSc [34, 35]. Similarly, RANKL was also an osteoclast-promoting mediator in periodontitis, which leads to periodontal bone resorption and tooth loss [36].

SSc is a systemic autoimmune disorder that could be substantially companied by psychology and neurological involvement [37]. Additionally, SSc decreased patients’ quality of life in many aspects, including feeling tired, sleep disorders, low self-esteem, as well as ineffective self-care [38]. In turn, the reduction in quality of life might interfere with the treatment and healing of SSc. Oral hygiene instruction and basic periodontal treatments are safe and sound medical choices to improve oral health and oral-related quality of life [39, 40]. Since periodontitis and SSc are both complex chronic diseases that shared similar pathology pathways, the improvement of oral health might contribute to optimal patient management in SSc treatment [41].

The present study is the first evidence-based analysis that quantitively evaluated the association between SSc and oral manifestations. Strengths of the present meta-analysis included the high quality of the eligible studies, with 8 out of 11 studies getting 7 or more NOS scores. The statement of PRISMA was strictly followed and the electronic databases were thoroughly searched. However, several limitations existed therefore the results should be interpreted with caution. First, the present study evaluated the existed literature regarding the association between SSc and oral manifestations. However, because of the rarity of SSc, the number of the included studies was relatively small, hence the publish bias was unable to be performed. Second, significant heterogeneities were observed, which might be caused by geographic and racial diversities. Moreover, all the included studies employed case-controlled designs, therefore the cause-effect relationship between SSc and periodontitis could not be perfectly elucidated.

## Conclusions

SSc is associated with oral pathological changes. SSc patients have limited mouth opening, higher periodontitis prevalence, and worse periodontal status, as well as an increased number of decayed teeth. Although further evidence is still needed, considering the etiological and pathological factors shared by SSc and periodontitis, the present study suggests clinicians be aware of oral hygiene and periodontal status in patients with SSc. Routinely oral hygiene instruction and initial periodontal treatment are recommended for SSc patients.

## Supplementary Information

**Additional file 1.** Search strategies.

## Data Availability

The readers can acquire available data and materials in the current study by sending an email to suyuwen1963@csu.edu.cn.

## Abbreviations

SSc: Systemic sclerosis
PD: Probing depth
AL: Attachment loss
BOP: Bleeding on probing
NOS: Newcastle-Ottawa Scale
M: Mean
SD: Standard deviation
SMD: Standard mean difference
CI: Confidence interval
OR: Odds ratio
OHRQoL: Oral Health Related Quality of Life
CRP: C-reactive protein
TNF: Tumor Necrosis Factor
IL: Interleukin
VEGF: Vascular endothelial growth factor
RANKL: Receptor activator for nuclear factor kappa beta ligand
